# Differences of Atomic-Level Interactions between Midazolam and Two CYP Isoforms 3A4 and 3A5

**DOI:** 10.3390/molecules28196900

**Published:** 2023-10-01

**Authors:** Shuhui Liu, Qingchuan Zheng, Fuquan Bai

**Affiliations:** 1 Institute of Theoretical Chemistry, College of Chemistry, Jilin University, Changchun 130023, China; baifq@jlu.edu.cn; 2 School and Hospital of Stomatology, Jilin University, Changchun 130023, China

**Keywords:** midazolam, CYP 3A4, CYP 3A5, molecular dynamic simulation, binding free energy, enzyme–substrate interaction

## Abstract

CYP 3A4 and CYP 3A5 are two important members of the human cytochrome P450 family. Although their overall structures are similar, the local structures of the active site are different, which directly leads to obvious individual differences in drug metabolic efficacy and toxicity. In this work, midazolam (MDZ) was selected as the probe substrate, and its interaction with two proteins, CYP 3A4 and CYP 3A5, was studied by molecular dynamics simulation (MD) along with the calculation of the binding free energy. The results show that two protein–substrate complexes have some similarities in enzyme–substrate binding; that is, in both complexes, Ser119 forms a high occupancy hydrogen bond with MDZ, which plays a key role in the stability of the interaction between MDZ and the enzymes. However, the complex formed by CYP 3A4 and MDZ is more stable, which may be attributed to the sandwich structure formed by the fluorophenyl group of the substrate with Leu216 and Leu482. Our study interprets the binding differences between two isoform–substrate complexes and reveals a structure–function relationship from the atomic perspective, which is expected to provide a theoretical basis for accurately measuring the effectiveness and toxicity of drugs for individuals in the era of precision medicine.

## 1. Introduction

Cytochrome P450 enzymes are found in cells throughout the body, but mainly in liver cells. Chemically, these enzymes catalyze the oxidation and metabolism of a wide range of exogenous and endogenous substances. Therefore, CYP450 enzymes are the main defense against exogenous substances and are responsible for the biological activation of drugs and toxins into more reactive intermediates. They also perform important endogenous functions. CYP450 has a highly reactive intermediate called Compound **1**. All endogenous and exogenous reactions are considered to be closely related to the reactivity of Compound **1**.

The cytochrome P450 3A subfamily (CYP 3A), accounting for more than 30% of liver CYP, has the largest content and substrate spectrum and hence is the most important rate-limiting enzyme in drug metabolism. The CYP 3A subfamily contains four members, including CYP 3A4, CYP 3A5, CYP 3A7, and CYP 3A43. CYP 3A4 is prominently expressed in adult liver, while CYP 3A5 is expressed in both fetal and adult liver [1]. In contrast, the contribution of CYP 3A7 and 3A43 to drug clearance in adults is negligible owing to their low expression in liver [2,3]. The expression and activity of CYP 3A4 are not affected by genetic factors, while CYP 3A5 is only expressed in about 20% of the population affected by the gene phenotype. At present, the research on CYP 3A subfamily often focuses on the selective metabolism of CYP 3A4, and the research on CYP 3A5 mainly focuses on genetic polymorphism and pharmacokinetics [4,5,6,7]. It is rare to compare the structural properties of the two isoforms after binding with the same ligand. In drug development, attention is often paid to the selective metabolism of CYP 3A4 [8,9]. The research on CYP 3A5 focuses on genetic polymorphism and pharmacokinetics [10,11]. The mechanism that determines the different catalytic efficiency of CYP 3A4 and CYP 3A5 has not been fully elucidated.

Many drugs can be metabolized by both CYP 3A4 and CYP 3A5, with different catalytic efficiency. An overlap for metabolic clearance by CYP 3A4 and 3A5 is evident for many drugs [4,5,6,7]. Studies on the development of a CYP 3A4 selective inhibitor and the vitro drug metabolism with 3A5 *1/*1 liver microsomes indicate that the contributions of CYP 3A4 and 3A5 to drug clearance are very different from each other [8]. Thereinto, CYP 3A5 has the highest drug metabolism efficiency [7]. J. Andrew Williams et al. demonstrated an equal or reduced metabolic capability for CYP 3A5 compared with CY P3A4 [9]. At this point, it is urgent to clarify the interaction between 3A4 or 3A5 and the same substrates at the atomic level, which will provide an important basis for understanding the mechanism of 3A4/3A5 metabolic clearance drugs.

Genetic factors have limited influence on the expression of CYP 3A4, and do not affect the activity of CYP 3A4 [8,9,10,11]. But the expression of CYP3A5 is closely related to the genetic phenotype [10,11,12]. CYP 3A5*1 alleles express higher levels of CYP 3A5 [1]. CYP 3A5*1 expression is highest in African ethnic groups [10]. Westlind-Johnsson et al. found that CYP3A5 in the Caucasian group has no significant effect on drug metabolism in liver [11]. The content of CYP 3A5 in the liver of the expressor accounts for more than half of the total CYP 3A subfamily. CYP 3A5 plays a crucial role in drug metabolism and is closely related to tumor chemoresistance [12]. The impact of genetic changes of the CYP 3A5 gene on CYP 3A5 activity in different ethnic groups may be the most important reason for individual and ethnic differences in CYP 3A drug metabolism.

**Figure 1:**
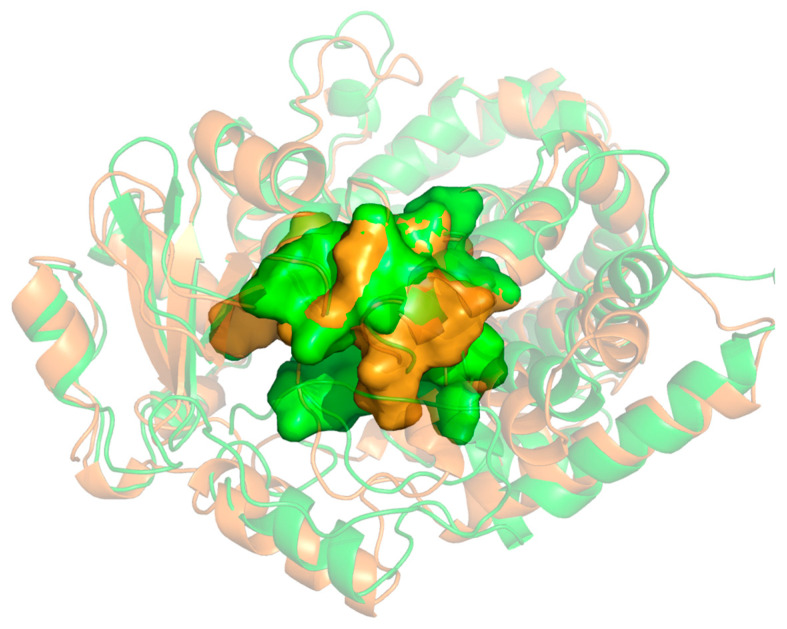
Structural comparison of two CYP isoforms, CYP 3A4 and CYP 3A5.

The secondary and tertiary structures of CYP 3A4 and CYP 3A5 are similar (Figure 1) (PDB ID: 5TE8 and 5VEU), and the amino acid sequences of 3A4 and 3A5 exhibit 83% sequence identity. Large active site cavity and flexibility are characteristics of CYP 3A4, which can bind molecules with large size span [8]. Mei Hui Hsu et al. determined the X-ray crystallography structure of CYP 3A5 and CYP 3A4 complexed with ritonavir, and found that the active site of CYP3A5 is taller and narrower than that of CYP 3A4 [13]. These two enzymes can catalyze reactions that form different metabolites from the same substrate [14,15]. However, the comparative study on the structure and properties of two subtypes binding with the same ligand is rare. In biological activities, the metabolism of same ligand is often a complex process involving various enzyme systems. A comparative study on the interaction mode, mechanism, and difference between CYP 3A4 and CYP 3A5 with the same ligand will provide an important theoretical basis for clinical individualized treatment.

**Figure 2:**
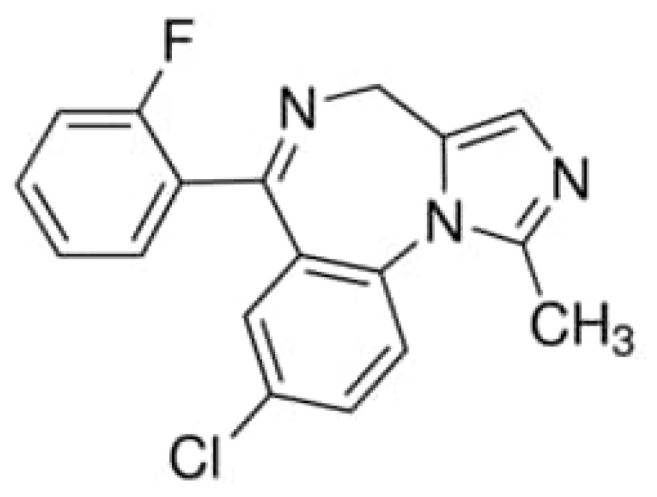
Chemical structure of midazolam.

Midazolam (MDZ) is the benzodiazepine most frequently used for procedural sedation (Figure 2), and is considered as a typical probe for the CYP 3A family [16,17,18]. The above information encourages us to use MDZ as a probe to investigate the similarities and differences between 3A4 and 3A5 interacting with the same substrate. To date, there has been a lot of computational work on CYP450s, particularly on the interactions between the enzymes and drug molecules [19,20,21,22,23,24,25,26]. In this work, molecular dynamics (MD) simulation was used to investigate the binding conformation, interaction analysis, and binding free energy of CYP 3A4 and CYP 3A5 with MDZ. We elucidated the interaction pattern and recognition mechanism of CYP 3A4 and CYP 3A5 with MDZ at the atomic level. The knowledge gained from our work might provide a theoretical basis for the accurate measurement of drugs for individuals with different genetic differential expression, and for the related research of drug effectiveness and toxicity.

## 2. Simulation Methods

### 2.1. Model Preparation 

The initial structure of CYP 3A4-midazolam complex (CYP 3A4-MDZ) was taken from Protein Data Bank (PDB ID: 5TE8). For the construction of the CYP 3A5-midazolam complex model, the X-ray diffraction crystal structure of CYP 3A5 combined with ritonavir (PDB ID: 5VEU) was selected as the pure protein structure of CYP 3A5, using Discovery Studio 3.1 to superimpose chain A of 5VEU and 5TE8 to determine the position of MDZ in CYP 3A5 [27,28]. Then, ritonavir in 5VEU was deleted, leading to a CYP 3A5-midazolam complex model (CYP 3A5-MDZ). The Modeller9.24 was used to supplement the missing residues in the two models [28]. Then, the MDZ molecule was optimized by Gaussian 09 software package under B3LPYP/6-31G functional theory. The force field parameters for the substrate MDZ were generated from the generalized amber force field (GAFF), following the RESP charge calculation in the antechamber module. On the basis of these two complex models, drug ligands were deleted to construct two pure protein models (APO-CYP 3A4 and APO-CYP 3A5), respectively. The four systems were solvated in a 10 Å truncated octahedron box using a TIP3P solvent mode under periodic boundary conditions. The H++ website was used to determine the protonation state of the titratable residues under the condition of pH = 7.0. An appropriate number of chlorine ions (Cl^−^), 1 for CYP 3A4 systems and 9 for CYP 3A5 systems, were incorporated into the systems to ensure the system neutrality.

### 2.2. Molecular Dynamics Simulation

MD simulation can provide detailed information on conformational changes at the atomic level, and has been widely used in the study of protein structure [19,20,21,22]. Compared with experimental techniques such as X-ray crystallography, which can only obtain static structural information of proteins, MD simulation can provide dynamic information of the structure–function relationship of cytochrome P450 [15,16,17,18]. We used the Amber16 software package to carry out classical MD simulations for four models, using the classical ff14sb force field [29,30]. The parameters of Cpd I were set according to Shahrokh’s work [31]. The force field of MDZ was generated by the antechamber program. The MD simulation process started with an energy minimization process, including 5000 steps of steepest descent method (SD) and 5000 steps of conjugate gradient method (CG). Then, each system was gradually heated from 0 K to 310 K, followed by a 500 ps equilibration process under the NVT ensemble. Finally, a 200 ns simulation of each system was carried out at 310 K under periodic boundary conditions in the NPT ensemble with 1 bar of pressure. Furthermore, two additional parallel simulations were performed to assess the rationality of the present simulations. The long-range electrostatic interactions were processed by the Particle–Mesh Ewald (PME) method [32]. The truncation distance for the local non-bonded interaction was set to 12 Å. SHAKE algorithm was used to constrain all bonds including hydrogen atoms. The integration time step was 2 fs [33]. PyMOL and VMD software were used to visualize the MD simulation trajectory and observe the details of the dynamic changes of the protein structure [34].

### 2.3. MM-PBSA Calculation 

The binding free energy calculation were carried out using the molecular mechanics of Possion–Boltzmann surface area (MM-PBSA) method implemented in Amber16. Estimation was based on the following equations:

Δ*G*_bind_ = *G*_complex_ − (*G*_receptor_ + *G*_ligand_) (1)

*G* = *E*_MM_ + *G*_sol_ – *T*Δ*S* (2)

*E*_MM_ = *E*_int_ + *E*_ele_ + *E*_vdw_ (3)

*G*_sol_ = *G*_PB_ + *G*_SA_ (4)

Δ*G*_SA_ = *γ*SASA+ *β* (5)

where *E*_MM_ is the gas phase molecular mechanical energy, *G*_sol_ is the solvation energy, and *E*_int_, *E*_ele_, and *E*_vdw_ represent internal energy, electrostatic energy, and van der Waals interaction energy, respectively. *G*_sol_ is composed of electrostatic solvation energy (*G*_PB_) and nonpolar solvation energy (*G*_SA_). *G*_sol_ is composed of electrostatic solvation energy (*G*_PB_) and nonpolar solvation energy (*G*_SA_). *T*Δ*S* is the entropy contribution. Here, Δ*G*_SA_ is calculated by solving Poisson–Boltzmann equation, whereas the latter is determined using Equation (5), where *γ* and *β* are two empirical constants and set to 0.00542 kcal mol^−1^ Å ^−2^ and 0.92 kcal mol^−1^, respectively. SASA is a solvent-accessible surface area (Å^2^) [35,36]. 

In order to understand the interaction between MDZ and two enzymes and find the residues that significantly contribute to binding, we used the MM/PBSA method to decompose the binding free energy into a single residue.

### 2.4. Analysis 

In the analysis of hydrogen bonds, the cutoff distance for the donor atom and the acceptor atom was set to 3.5 Å, and the angle of the donor, hydrogen, and the acceptor was set to 120°. The occupancy rate of hydrogen bond is the proportion of frames forming hydrogen bonds in the MD trajectory. The criterion for salt bridge is that the distance is less than 4.5 Å.

## 3. Result and Discussion

### 3.1. Analysis of Enzyme-Substrate Binding Pattern

**Figure 3:**
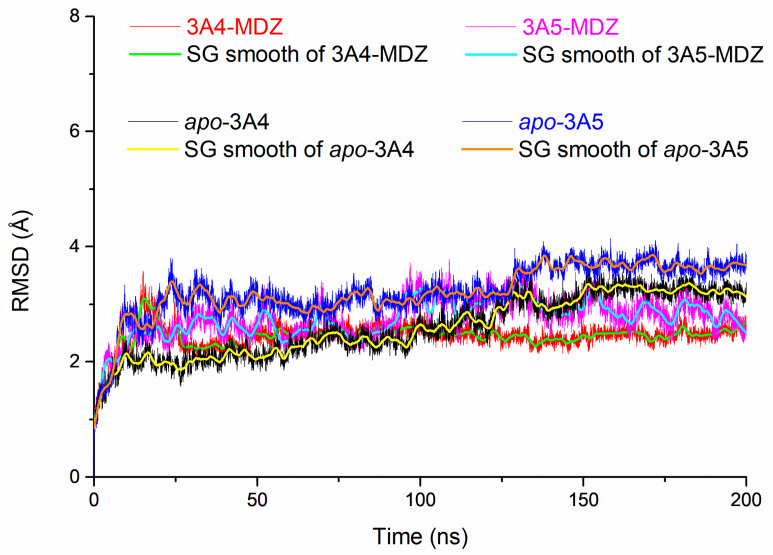
Root-mean-square deviation (RMSD) versus simulation of time.

To examine the convergence of the systems, the root-mean-square deviations (RMSD) of all the backbone atoms for four complex structures were calculated (see Figure 3 and Figures S1–S4). The RMSD values of all the trajectories of the four systems in the last 100 ns fluctuate less than 4 Å, which indicates that the four systems have reached conformational stability and maintain equilibrium. In order to reduce the influence of the initial structure on the conformation, we chose the last 100 ns simulation trajectory to analyze the conformational characteristics of the system.

**Figure 4:**
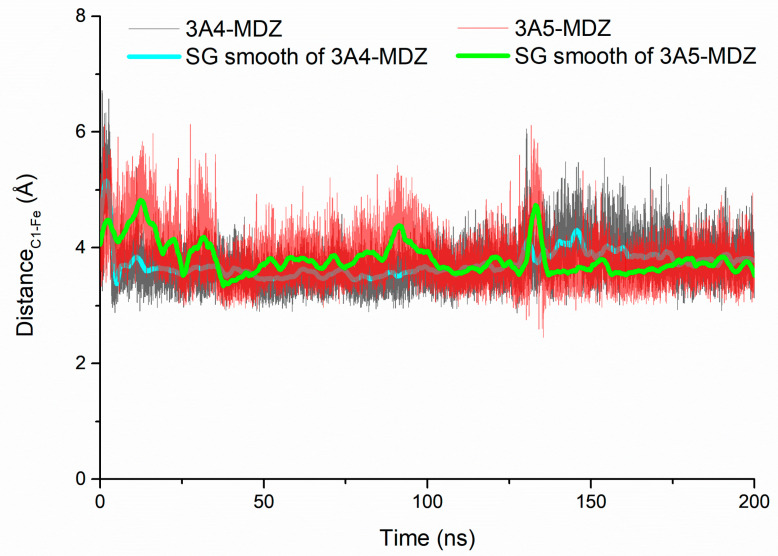
Distance between MDZ-C1 and Fe atoms in porphyrin rings of CYP3A4 (black) and CYP3A5 (red).

The catalytic efficiency of cytochrome P450 is dependent on the distance of hydroxylated carbon from the heme iron. A less favorable distance results in less productive catalysis [37]. If the distance between a carbon atom and the heme iron is within 5 Å, the substrate is likely to be hydroxylated; i.e., the distance is a prerequisite for hydroxylation. [38]. In this regard, we monitored the distance between the hydroxylated carbon atom (C1) of the substrate and heme iron atom in the two complexes in the 200 ns production simulations. As shown in Figure 4, the distance between the iron atom on the porphyrin ring and the C1 atom of the substrate MDZ in the two complex structures is highly concentrated at 3.5 Å, both within the reaction distance range (5 Å), which proves that MDZ can be catalyzed by CYP3A4 and CYP3A5.

**Figure 5:**
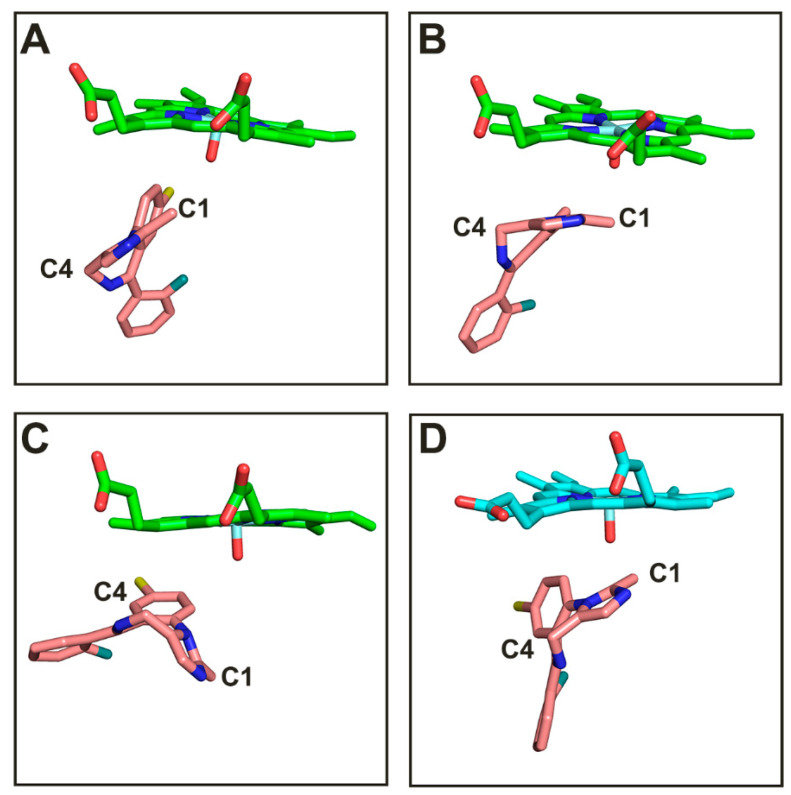
Representative binding conformations of MDZ in CYP3A4 (**A**–**C**) and CYP3A5 (**D**).

In the current work, multiple replications of the 200 ns MD simulation were performed and the production simulation were monitored. According to the change in the distances between the MDZ oxidation sites (C1 and C4) and heme iron, substrate-binding conformations were identified. There are three types of complex conformations for CYP3A4-MDZ and only one type for CYP3A5-MDZ. The representative conformations of CYP3A4-MDZ named poses A, B, C, and the conformations of CYP3A5-MDZ named pose D are showed in Figure 5. In pose A and C, the C1 and C4 of MDZ are not at the same horizon parallel to the heme iron. The distance between C1 of MDZ and heme iron is closer in pose A, while the C4 group faces the heme iron. In pose B, the C1 and C4 atoms of MDZ are almost at the same horizon parallel to the heme. In the conformation of CYP3A5-MDZ, the distance between the C1 atom of MDZ and heme iron is less than that between the C4 atom and heme iron.

### 3.2. Factors That Stabilize Substrate Binding

**Figure 6:**
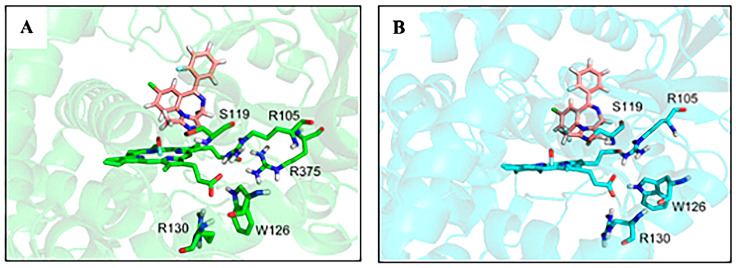
Hydrogen bonding at the active site of two complex systems, (**A**) CYP3A4 and (**B**) CYP3A5.

Hydrogen bonds play a key role in the structural stability of protein. It can be clearly seen that only one hydrogen bond forms between the substrate MDZ and the protein in each of two complex systems, and both of them exist between the N2 atom of the substrate and the residue Ser119 (Figure 6). There is very high occupancy rate of the hydrogen bond in both of the two complex, 93.8% (CYP3A4-MDZ) and 77.9% (CYP3A5-MDZ), respectively (Table 1). Sevrioukova et al. observed that the hydrogen bond formed with Ser119 in CYP3A4-MDZ is the key to achieve the MDZ binding pattern [39]. Khan, Roussel et al. also confirmed that Ser119 is the key active site residue that determines the substrate binging affinity, orientation, and oxidation site in the metabolic study of the CYP3A4-MDZ complex [40,41]. We reached the same conclusion as the experiment in [39]: that is, Ser119 plays an important role in the stability of substrate and enzyme binding in CYP3A4-MDZ system. At the same time, we also found that Ser119 plays an important role in the stability of substrate and enzyme binding in the CYP3A5-MDZ system.

Similar hydrogen bond networks were observed between porphyrin rings and surrounding amino acid residues in both systems. O1 and O2 atoms of the porphyrin ring form hydrogen bonds with Arg130, Arg105, and Trp126 in two isoforms. (Figure 6, Table 1) The heme group forms only one hydrogen bond with the residue Arg130 in each complex system. But the occupancy of the hydrogen bond in the CYP3A4-MDZ complex (94.0%) is significantly higher than that in the CYP3A5-MDZ complex (50.7%). The total occupancy of hydrogen bonds with the residue Trp126 in two complexes is similar, 168.0% (92.5% + 75.5%) and 176.2% (90.7% + 85.5%), respectively. The residue Arg105 behaves similarly to the residue Trp126 in two isoforms. In CYP3A4-MDZ complex, the cofactor heme forms three hydrogen bonds with the residue Arg105, the total occupancy is 226.4% (65.1% + 83.9% + 77.4%). While in CYP3A5-MDZ complex, the cofactor heme forms six hydrogen bonds with the residue Arg105, the total occupancy is 263.6% (68.4% + 52.1% + 43.4% + 34.8% + 31.4% + 33.5%). Furthermore, the heme group in the CYP3A4-MDZ system forms four hydrogen bonds with the residue Arg375, and the occupancy rate are all very high. It can be seen that the heme group has more hydrogen bond interaction in the CYP3A4-MDZ system than in CYP3A5-MDZ. Hydrogen bond interaction is a key factor to stabilize substrate binding and complex structure. These hydrogen bonds between the substrate MDZ and amino acid residues along with the hydrogen bond network formed by the iron porphyrin ring and amino acid residues, help anchor the position of porphyrin ring and keep the porphyrin ring and MDZ within the reaction distance, facilitating the hydroxylation of MDZ-C1 atoms in a proper position. Compared with CYP3A5-MDZ complex, CYP3A4-MDZ structure has a higher and more stable hydrogen bond network, indicating that CYP3A4 binds to substrates more stably in the same environment, which is more conducive to the maintenance of protein activity. The parallel simulations give the similar data. (Tables S1–S4) We speculate that the active site of CYP3A4 is suitable for the binding of MDZ. 

In addition to hydrogen bonding, we also monitored the salt bridge network in the complex systems (Table 2). Stable salt bridge networks exist in both CYP3A4-MDZ and CYP3A5-MDZ complexes. It can be seen in Table 2 that the residues Arg130 mentioned above form the salt bridge with the residue Asn441 in the CYP3A4-MDZ complex. At the same time, the residue Arg106 has the salt bridge network with the residue Glu374 in the CYP3A4-MDZ complex. In the CYP3A5-MDZ complex, there is only one salt bridge that forms between the residues Arg106 and Glu374. These salt bridge networks stabilize the hydrogen bond network in the complex, which help anchor the position of the porphyrin ring and keep it within the reaction distance of MDZ. The difference of the salt bridge effect between the two complex systems may be a manifestation of the difference of substrate and enzyme binding modes.

**Figure 7:**
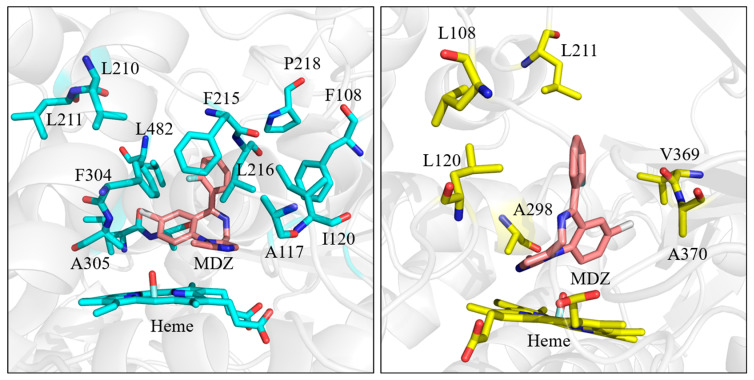
Hydrophobic interaction in the binding site of CYP3A4-MDZ (**left**) and CYP3A5-MDZ (**right**).

**Figure 8:**
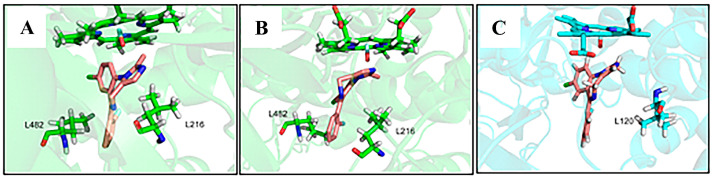
Binding conformation of MDZ in the CYP3A4-MDZ complex conformation (**A**,**B**) and in the CYP3A5-MDZ complex (**C**).

Except the hydrogen bond and salt bridge interaction, hydrophobic interaction is also very important in the process of binding enzymes to ligands. During the molecular dynamics simulation, it was observed that MDZ is located in a binding pocket composed of 11 residues (Phe108, Ala117, Ile120, Phe215, Leu216, Pro218, Phe304, Ala305, Ile369, Ala370, Leu482) in the CYP3A4-MDZ system, and located in a binding pocket composed of five residues (Leu120, Leu221, Ala298, Val369, Ala370) in the CYP3A4-MDZ system (Figure 7). All the residues are non-polar residues, creating a hydrophobic environment for MDZ binding. As well as in the CYP3A4-MDZ system, the fluorophenyl group is sandwiched between Leu216 and Leu482. This sandwich structure restricts rotation, lateral and vertical motion of MDZ, and immobilized it in a position suitable for C1 atom hydroxylation. However, the fluorophenyl group of CYP3A5-MDZ system cannot form a sandwich structure with the nearby residues. Leu120 is the only residue nearby and the other side of the fluorophenyl group is exposed in the cavity (Figure 8), which may be related to the fact that the active pocket of CYP3A5 is higher and narrower than that of CYP3A4 [13].

The conformational difference of MDZ in the binding pockets of two enzymes reflects the difference of the local amino acids [13]. The residue Lys216 in the CYP3A5-MDZ system is different from Leu216 of CYP3A4, and Lys216 is positively charged. The narrow cavity bottom and the difference in the local amino acid residues may be the reason why MDZ fluorophenyl does not form a sandwich structure when it binds to CYP3A5. The lack of this sandwich structure in CYP3A5-MDZ system may affect the stability of the substrate binding to the protein, and subsequently affect the metabolite profile and metabolic efficiency.

The strength of hydrophobicity was evaluated on the basis of the Expasy score (https://web.expasy.org/protscale/, accessed on 10 September 2022). The higher the score, the stronger the hydrophobicity. The scores of the hydrophobic interactions in two complex systems are listed in Table 3. As shown in the table, although the hydrophobic residues in both complexes form pockets to wrap the substrate, which plays an important role in the stability between the substrate and the enzyme, the number of amino acids involved in hydrophobic interaction near the active site of CYP3A4-MDZ complex is more, and the interaction is stronger and more stable than that of CYP3A5-MDZ. The sandwich structure formed by fluorophenyl, Leu216, Leu482 in CYP3A4-MDZ system and the hydrophobic interaction with higher score near the active site both indicates that the binding of MDZ and CYP3A4 is more stable. The above hydrophobic scores keep the consistency with the contact number analysis in two parallel MD simulations (Table S5).

### 3.3. Binding Free Energy Analysis 

To get insight into the interactions between the ligand and the enzyme, binding free energy of two complex systems was calculated by the MM-PB/SA method in Amber16, and the contribution of entropy was also considered [42,43]. An amount of 5000 frames is uniformly selected from the last 100 ns trajectory of equilibrium for calculation, and the results are listed in Table 4. For better understanding the binding details between enzyme and ligand, the binding free energies were decomposed into each residue, and the results are listed in Table 5 and Table 6, respectively. The results showed that the binding free energies between MDZ and CYP3A4 and CYP3A5 are −19.5 kcal/mol and −11.6 kcal/mol, respectively, indicating that the binding between CYP3A4 and MDZ is more stable than CYP3A5. A detailed energy analysis shows that the polar interaction energies are similar in the two complex systems. The electrostatic interaction is conducive to binding, but its contribution is offset by the greater polar solvation, so the non-polar interaction is the major driving force for the substrate–enzyme binding.

As described in the hydrogen bond interaction, the occupancy of hydrogen bond between substrate MDZ and Ser119 and the total occupancy of hydrogen bonds with Arg105 in CYP3A5-MDZ system are slightly higher than those in CYP3A4-MDZ system. Especially the occupancy of hydrogen bond formed by Ser119 has no significant difference. The same results are shown in the residue decomposition energy. Ser119 contributes −2.20 ± 0.59 kcal/mol (CYP3A4-MDZ) and −2.49 ± 0.52 kcal/mol (CYP3A5-MDZ) to binding MDZ in two complex systems, respectively. Arg105 contributes −0.66 ± 0.41 kcal/mol (CYP3A4-MDZ) and −0.74 ± 0.37 kcal/mol (CYP3A5-MDZ) for stabilizing the conformation, respectively. Although the energy contribution of Ser119 and Arg105 in CYP3A5-MDZ system is similar, the electrostatic energy of CYP3A4-MDZ system is more conducive to binding. In addition, the results of residue decomposition energy clearly indicate that the contributions of some hydrophobic amino acids to the system are significantly (such as Leu216, Leu482, Ala 370, Phe 304, Ile 369, Arg105, Ala305 in CYP3A4-MDZ system. Arg105, Leu211, Ala370, Val369, Ala298 in CYP3A5-MDZ system). MDZ is surrounded by hydrophobic cavities formed by these hydrophobic amino acids. In particular, Leu216 (−2.67 ± 0.64 kcal/mol) and Leu482 (−1.45 ± 0.34 kcal/mol) clip fluorophenyl in the middle to form a sandwich structure in CYP3A4-MDZ system. These amino acids, forming strong hydrophobic interaction with MDZ, play an important role in stabilizing the binding of substrates, and explain why nonpolar interaction is the main driving force.

## 4. Conclusions

In this work, we obtained different conformations of CYP3A4-MDZ and CYP3A5-MDZ. The residue Ser119 of CYP3A4 and CYP3A5 forms a high occupancy hydrogen bond with MDZ, which plays an important role in the stability of the interaction between substrate MDZ and enzyme. The hydrogen bond network and salt bridge network near the active site of CYP3A4-MDZ and CYP3A5-MDZ are rich and with high occupancy. These hydrogen bonds and salt bridge structures play an important role in anchoring the iron porphyrin ring and ensuring the interaction distance between the iron porphyrin ring and MDZ. The presence of these actions and structures makes the substrate MDZ at a relatively constant position at the active site for efficient reaction. The analysis of hydrophobic interactions shows that it is stronger and more stable in CYP3A4-MDZ. Although the residues involved in hydrophobic interactions in the two complexes are different, they all form pocket to wrap the substrate. These residues make dominant contributions for the stability between substrate and enzyme. In addition, we found fluorophenyl was sandwiched in Leu216 and Leu482 in CYP3A4-MDZ complex, but there was no such structure in CYP3A5-MDZ complex. By comparing the results of conformational state, hydrogen bond interaction, hydrophobic interaction, electrostatic interaction, binding free energy, and decomposition energy, we found that both CYP3A4 and CYP3A5 could bind to the substrate MDZ (C1) and the structure of CYP3A4-MDZ is more stable. The differences are described at the atomic level about CYP3A4 and CYP3A5 metabolizing the same substrate, which will help to design precise measurement drugs for individuals with different genetic differential expression, and provide theoretical basis for the related research on drug effectiveness and toxicity.

## Figures and Tables

**Table 1:** Occupancy of the H-bonds for two complex systems in the production simulations.

Complex	The Recipient and the Donor	Ratio (%)
CYP3A4-MDZ	heme@O1A-105R@HH21-NH2	65.1
	heme@O2D-105R@HH22-NH2	83.9
	heme@O2D-105R@HH12-NH1	77.4
	heme@O1D-126W@HE1-NE1	92.5
	heme@O2D-126W@HE1-NE1	75.5
	heme@O1D-130R@NH11-NH1	94.0
	heme@O1A-375R@NH12-NH1	76.5
	heme@O1A-375R@NH22-NH2	86.6
	heme@O2A-375R@NH22-NH2	89.5
	heme@O2A-375R@NH12-NH1	52.9
	MDZ@NAN-119S@HG-OG	93.8
CYP3A5-MDZ	heme@O1D-105R@HH22-NH2	68.4
	heme@O2A-105R@HH21-NH2	52.1
	heme@O1A-105R@HH21-NH2	43.4
	heme@O2A-105R@HE-NE	34.8
	heme@O1A-105R@HE-NE	31.4
	heme@O2D-105R@HH22-NH2	33.5
	heme@O1D-126W@HE1-NE1	90.7
	heme@O2D-126W@HE1-NE1	85.5
	heme@O2D-130R@NH11-NH1	50.7
	MDZ@NAN-119S@HG-OG	77.9

**Table 2:** Salt bridge in the complex systems in the production simulations.

Complex	Salt Bridge Action	Ratio (%)
CYP3A4-MDZ	374E@OE1-106R@HH12-NH1	67.30
	374E@OE1-106R@HH11-NH1	67.30
	374E@OE2-106R@HH12-NH1	61.57
	374E@OE2-106R@HH11-NH1	61.57
	374E@OE2-106R@HE-NE	43.03
	374E@OE1-106R@HE-NE	54.53
	441N@OD1-130R@HH22-NH2	96.40
	441N@OD1-130R@HH21-NH2	96.40
	441N@OD1-130R@HH12-NH1	89.17
	441N@OD1-130R@HH11-NH1	89.17
	441N@ND2-130R@HH22-NH2	37.70
	441N@ND2-130R@HH21-NH2	37.70
CYP3A5-MDZ	76E@OE1-106R@HH12-NH1	87.37
	76E@OE1-106R@HH11-NH1	87.37
	76E@OE1-106R@HH22-NH2	88.67
	76E@OE1-106R@HH21-NH2	88.67
	76E@OE2-106R@HH12-NH1	79.87
	76E@OE2-106R@HH11-NH1	79.87
	76E@OE2-106R@HH22-NH2	64.03
	76E@OE2-106R@HH21-NH2	64.03
	374E@OE2-106R@HH12-NH1	33.97
	374E@OE2-106R@HH11-NH1	33.97
	374E@OE1-106R@HH12-NH1	32.73
	374E@OE1-106R@HH11-NH1	32.73

**Table 3:** Hydrophobic scores of two complex systems.

Complex	Residue	Score
CYP3A4-MDZ	Phe108	−1.056
	Ala117	1.311
	Ile120	0.056
	Phe215	0.322
	Leu216	0.211
	Pro218	0.733
	Phe304	1.744
	Ala305	1.167
	Ile369	0.889
	Ala370	1.000
	Leu482	0.133
CYP3A5-MDZ	Leu120	−0.022
	Leu211	−0.067
	Ala297	1.167
	Val369	1.144
	Ala370	1.256

**Table 4:** The binding free energy of two complex systems (kcal/mol).

	3A4-MDZ	3A5-MDZ
∆*E*_ele_	−15.5 ± 2.5	−12.0 ± 2.7
∆*E*_vdw_	−38.3 ± 2.7	−30.5 ± 2.2
∆*G*_PB_	23.5 ± 1.5	19.9 ± 1.8
∆*G*_SA_	−4.3 ± 0.2	−3.3+0.2
∆*G*_gas_	−53.8 ± 3.5	−42.5 ± 3.6
∆*G*_solv_	19.2 ± 1.5	16.6 ± 1.7
^a^ ∆*G*_pol_	8.00	7.95
^b^ ∆*G*_nonpol_	−43.6	−33.8
^c^ ∆*G*_MMPB/SA_	−34.6 ± 2.9	−25.8 ± 2.5
*T*∆*S*	−15.1 ± 7.4	−14.2 ± 5.5
^d^ ∆*G*_bind_	−19.5	−11.6

^a^ *∆G*_pol_ = *∆E*_ele_ + *∆G*_PB_; ^b^ **∆***G*_nonpol_ = **∆***E*_vdw_ + **∆***G*_SA_; ^c^ **∆***G*_MM-PB/SA_ = **∆***E*_ele_ + **∆***E*_vdw_ + **∆***G*_PB_ + **∆***G*_SA_; ^d^ **∆***G*_bind_ = **∆***G*_MM-PB/SA_ − *T***∆***S.*

**Table 5:** Decomposition energy of key amino acid residues in CYP3A4-MDZ (kcal/mol).

Residue	∆*E*_vdw_	∆*E*_ele_	∆*G*_PB_	∆*G*_SA_	∆*G*_bind_
Leu216	−0.77 ± 0.57	−0.15 ± 0.27	0.61 ± 0.30	−0.36 ± 0.06	−2.67 ± 0.64
Ser119	−0.28 ± 0.58	−3.74 ± 0.98	1.90 ± 0.28	−0.09 ± 0.03	−2.20 ± 0.59
Leu482	−1.29 ± 0.31	−0.12 ± 0.07	0.19 ± 0.05	−0.23 ± 0.06	−1.45 ± 0.34
Ala370	−1.17 ± 0.26	−0.03 ± 0.05	0.19 ± 0.08	−0.23 ± 0.05	−1.23 ± 0.27
Phe304	−1.10 ± 0.38	−0.07 ± 0.07	0.37 ± 0.14	−0.16 ± 0.04	−0.96 ± 0.32
Ile369	−0.70 ± 0.27	−0.08 ± 0.07	0.05 ± 0.07	−0.05 ± 0.02	−0.78 ± 0.27
Arg105	−0.64 ± 0.31	−1.43 ± 0.42	1.47 ± 0.40	−0.07 ± 0.03	−0.66 ± 0.41
Asp217	−0.83 ± 0.23	−1.13 ± 0.34	1.54 ± 0.46	−0.12 ± 0.03	−0.54 ± 0.23
Ile301	−0.51 ± 0.13	−0.17 ± 0.09	0.21 ± 0.09	−0.03 ± 0.01	−0.50 ± 0.13
Thr309	−0.43 ± 0.13	−0.08 ± 0.10	−0.08−0.08	−0.05 ± 0.02	−0.50 ± 0.13
Ala305	−0.41 ± 0.18	−0.09 ± 0.07	−0.07−0.05	−0.03 ± 0.02	−0.42 ± 0.17
HEM	−5.12 ± 0.63	−0.88 ± 0.56	1.72 ± 0.42	−0.35 ± 0.04	−4.62 ± 0.76

**Table 6:** Composition energy of key amino acid residues in CYP3A5-MDZD (kcal/mol).

Residue	∆*E*_vdw_	∆*E*_ele_	∆*G*_PB_	∆*G*_SA_	∆*G*_bind_
Ser119	−0.71 ± 0.65	−3.78 ± 0.84	2.15 ± 0.30	−0.15 ± 0.04	−2.49 ± 0.52
Thr302	−1.68 ± 0.45	−0.21 ± 0.22	0.45 ± 0.21	−0.26 ± 0.04	−1.70 ± 0.49
Phe297	−1.36 ± 0.47	−0.36 ± 0.16	0.68 ± 0.21	−0.19 ± 0.05	−1.23 ± 0.43
Arg105	−0.68 ± 0.19	−0.49 ± 0.44	0.48 ± 0.39	−0.05 ± 0.02	−0.74 ± 0.37
Leu211	−0.55 ± 0.21	−0.08 ± 0.05	0.18 ± 0.07	−0.18 ± 0.05	−0.63 ± 0.23
Ala370	−0.50 ± 0.23	−0.02 ± 0.07	0.16 ± 0.10	−0.14 ± 0.05	−0.50 ± 0.24
Val369	−0.51 ± 0.22	−0.02 ± 0.18	0.14 ± 0.20	0.07 ± 0.03	−0.47 ± 0.24
Ala298	−0.44 ± 0.14	−0.08 ± 0.05	0.16 ± 0.08	−0.02 ± 0.01	−0.39 ± 0.15
HEM	−5.12 ± 0.75	−1.29 ± 0.69	2.48 ± 0.88	−0.39 ± 0.05	−4.32 ± 0.70

## Data Availability

All the simulation results and data can be directed to the corresponding author.

## Supplementary Materials

The following supporting information can be downloaded at: https://www.mdpi.com/article/10.3390/molecules28196900/s1, Figure S1: RMSD plots of protein heavy atoms, the residues in the binding site, and the ligand in two additional parallel MD simulations for CYP3A4-MDZ system; Figure S2: RMSD plots of protein heavy atoms in two additional parallel MD simulations for *apo*-CYP3A4 system; Figure S3: RMSD plots of protein heavy atoms, the residues in the binding site, and the ligand in two additional parallel MD simulations for CYP3A5-MDZ system; Figure S4: RMSD plots of protein heavy atoms in two additional parallel MD simulations for *apo*-CYP3A5 system; Figure S5: Molecular dynamics simulations reveal the different interaction of midazolam with two cytochrome P450 proteins, CYP 3A4 and CYP 3A5; Table S1: Hydrogen bond percentage in CYP3A4-MDZ complex in two parallel simulations; Table S2: Hydrogen bond percentage in CYP3A5-MDZ complex in two parallel simulations; Table S3: Salt bridge percentage in CYP3A4-MDZ complex in two parallel simulations; Table S4: Salt bridge percentage in CYP3A5-MDZ complex in two parallel simulations; Table S5: The contact number between MDZ and the active residues of the isoforms (5 Å near the active site) in two parallel simulations.
