# Supplementary material for: Differences of Atomic-Level Interactions between Midazolam and Two CYP Isoforms 3A4 and 3A5
Source: Molecules. 2023 Oct 1;28(19):6900. doi: 10.3390/molecules28196900 (PMC10574787; doi:10.3390/molecules28196900)
Supplement: Supplementary file 1 [file molecules-28-06900-s001.zip › molecules-2534716-supplementary.pdf]

Differences of atomic-level interactions between midazolam and two CYP isoforms 3A4 and 3A5

Shuhui Liu[1,2,*], Qingchuan Zheng[1,*], and Fuquan Bai[1]

[1] *Institute of Theoretical Chemistry, College of Chemistry, Jilin University, Changchun 130023, Jilin, People's Republic of China*

[2] *School and Hospital of Stomatology, Jilin University, Changchun 130023, Jilin, People's Republic of China*

*To whom correspondence should be addressed. E-mail: serene@jlu.edu.cn; zhengqc@jlu.edu.cn.

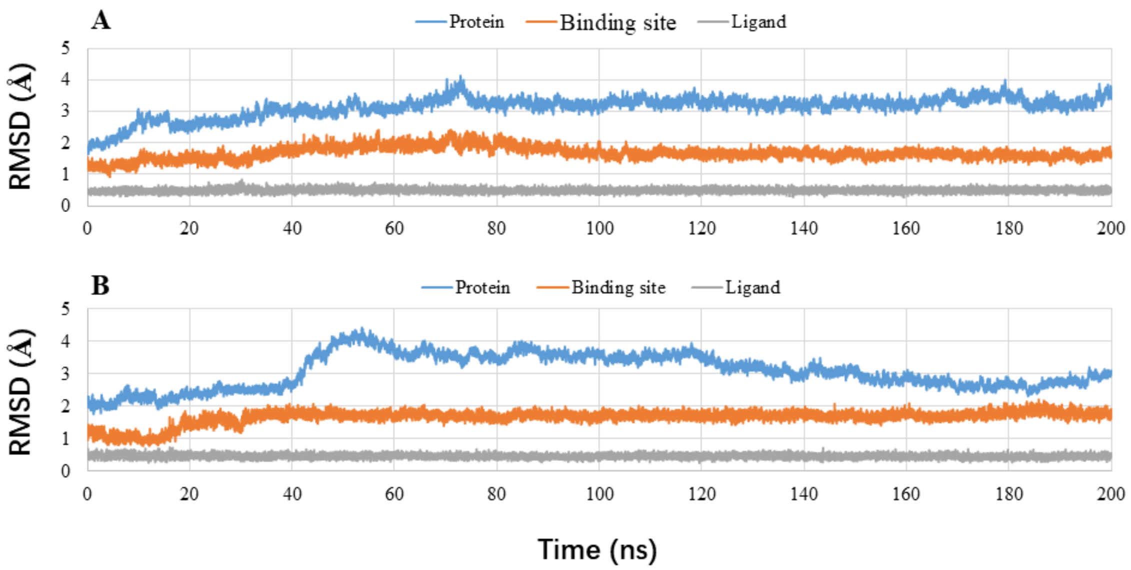

Figure S1. RMSD plots of protein heavy atoms, the residues in the binding site, and the ligand in two additional parallel MD simulations for CYP3A4-MDZ system.

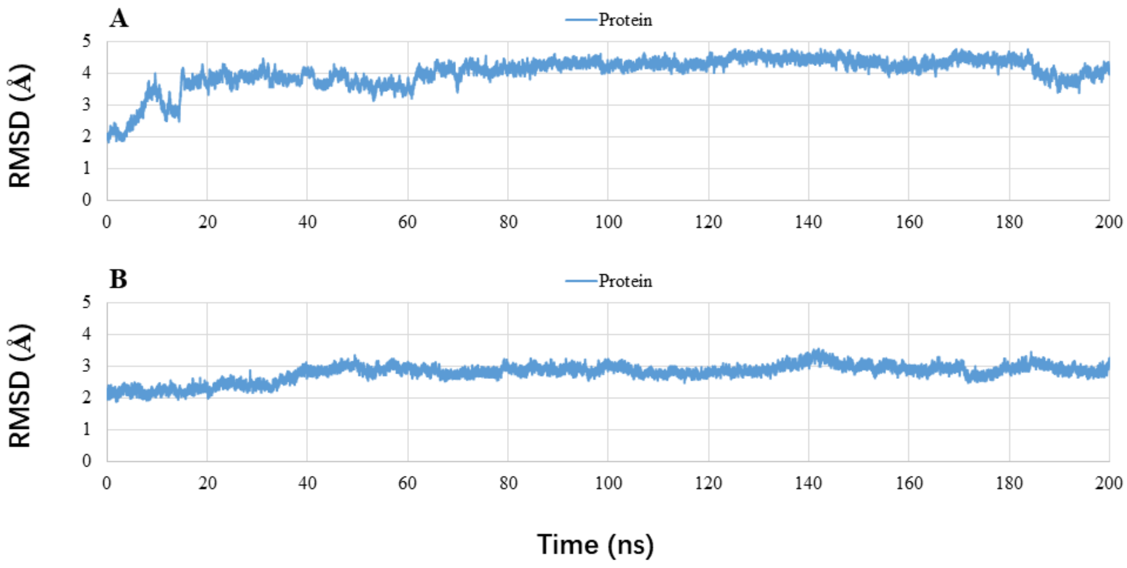

Figure S2. RMSD plots of protein heavy atoms in two additional parallel MD simulations for *apo*-CYP3A4 system.

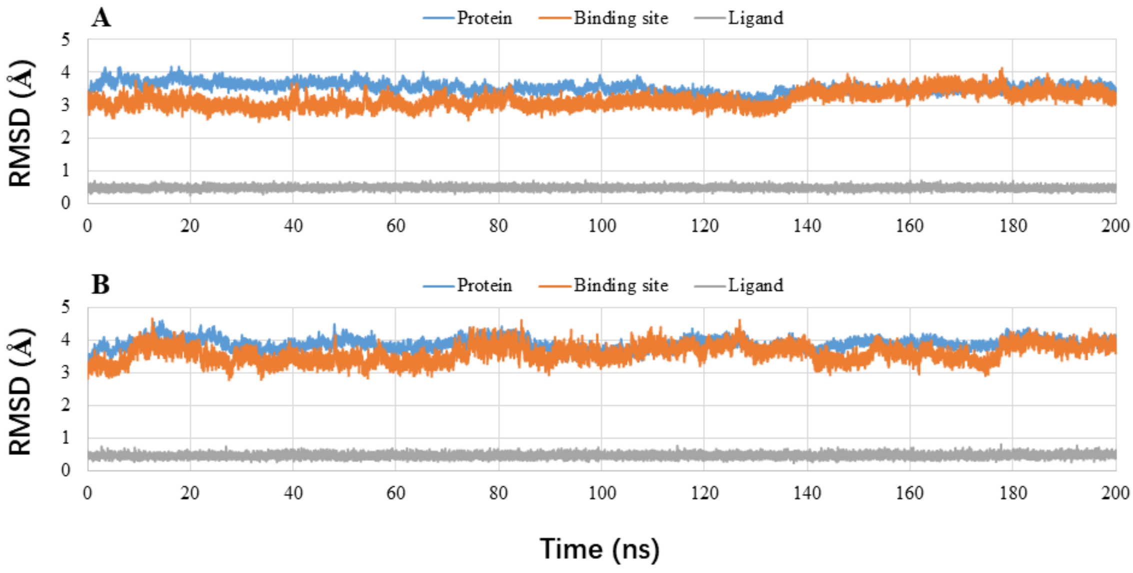

Figure S3. RMSD plots of protein heavy atoms, the residues in the binding site, and the ligand in two additional parallel MD simulations for CYP3A5-MDZ system.

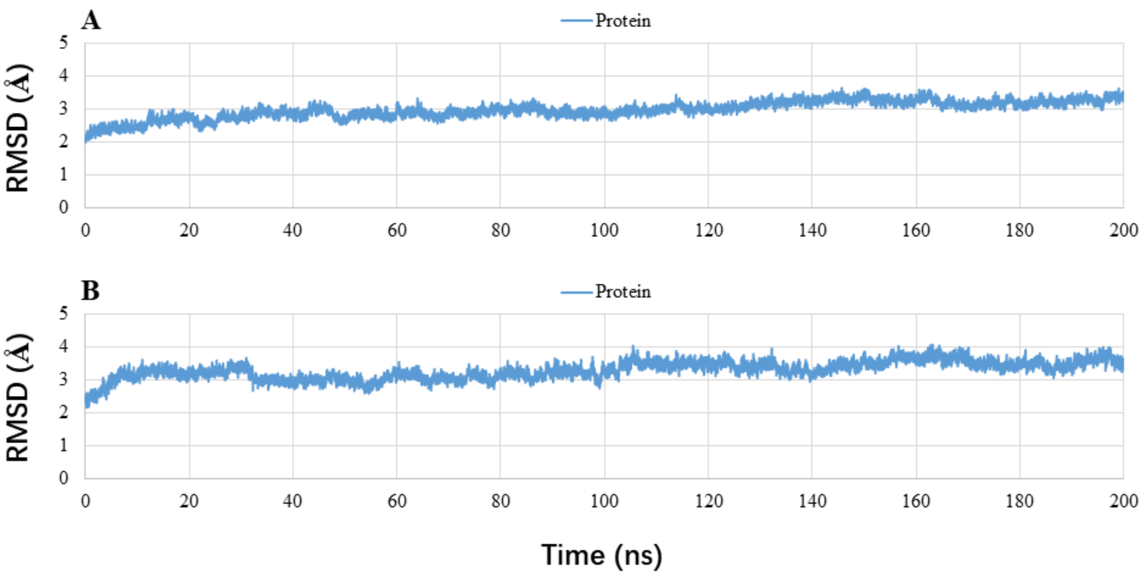

Figure S4. RMSD plots of protein heavy atoms in two additional parallel MD simulations for *apo*-CYP3A5 system.

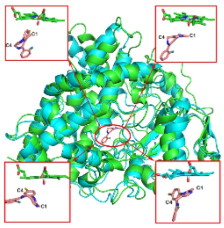

Figure S5. Molecular dynamics simulations reveal the different interaction of midazolam with two cytochrome P450 proteins, CYP 3A4 and CYP 3A5.

Table S1. Hydrogen bond percentage in CYP3A4-MDZ complex in two parallel simulations.

Complex CYP3A4-MDZ	The recipient and the donor	ratio
System A	heme@O1A-105R@HH21-NH2	69.20
	heme@O2D-105R@HH22-NH2	80.40
	heme@O2D-105R@HH12-NH1	75.90
	heme@O1D-126W@HE1-NE1	87.20
	heme@O2D-126W@HE1-NE1	75.00
	heme@O1D-130R@NH11-NH1	90.40
	heme@O1A-375R@NH12-NH1	74.90
	heme@O1A-375R@NH22-NH2	87.20
	heme@O2A-375R@NH22-NH2	92.4
	heme@O2A-375R@NH12-NH1	50.30
	MDZ@NAN-119S@HG-OG	93.50

System B	heme@O1A-105R@HH21-NH2	63.40
	heme@O2D-105R@HH22-NH2	83.00
	heme@O2D-105R@HH12-NH1	80.80
	heme@O1D-126W@HE1-NE1	94.10
	heme@O2D-126W@HE1-NE1	72.80
	heme@O1D-130R@NH11-NH1	91.80
	heme@O1A-375R@NH12-NH1	75.70
	heme@O1A-375R@NH22-NH2	85.40
	heme@O2A-375R@NH22-NH2	87.20
	heme@O2A-375R@NH12-NH1	50.90
	MDZ@NAN-119S@HG-OG	92.6

Table S2. Hydrogen bond percentage in CYP3A5-MDZ complex in two parallel simulations.

Complex CYP3A5-MDZ	The recipient and the donor	ratio
System A	heme@O1D-105R@HH22-NH2	69.5
	heme@O2A-105R@HH21-NH2	50.40
	heme@O1A-105R@HH21-NH2	43.00
	heme@O2A-105R@HE-NE	34.20
	heme@O1A-105R@HE-NE	31.30
	heme@O2D-105R@HH22-NH2	30.50
	heme@O1D-126W@HE1-NE1	93.60
	heme@O2D-126W@HE1-NE1	84.70
	heme@O2D-130R@NH11-NH1	53.87
	MDZ@NAN-119S@HG-OG	57.20
System B	heme@O1D-105R@HH22-NH2	65.36
	heme@O2A-105R@HH21-NH2	52.10
	heme@O1A-105R@HH21-NH2	43.40
	heme@O2A-105R@HE-NE	32.62
	heme@O1A-105R@HE-NE	29.54
	heme@O2D-105R@HH22-NH2	36.67
	heme@O1D-126W@HE1-NE1	84.00
	heme@O2D-126W@HE1-NE1	88.70
	heme@O2D-130R@NH11-NH1	45.74
	MDZ@NAN-119S@HG-OG	78.23

Table S3. Salt bridge percentage in CYP3A4-MDZ complex in two parallel simulations.

Complex CYP3A4-MDZ	The recipient and the donor	ratio
System A	374E@OE1-106R@HH12-NH1	86.10
	374E@OE1-106R@HH11-NH1	86.10
	374E@OE2-106R@HH12-NH1	70.40
	374E@OE2-106R@HH11-NH1	70.40
	374E@OE2-106R@NE-NE	49.00
	374E@OE1-106R@NE-NE	77.80
	441N@OD1-130R@HH22-NH2	99.60
	441N@OD1-130R@HH21-NH2	99.60
	441N@OD1-130R@HH12-NH2	NA
	441N@OD1-130R@HH11-NH1	NA
	441N@ND2-130R@HH22-NH2	19.30
	441N@ND2-130R@HH21-NH2	19.30
System B	374E@OE1-106R@HH12-NH1	NA
	374E@OE1-106R@HH11-NH1	NA
	374E@OE2-106R@HH12-NH1	NA
	374E@OE2-106R@HH11-NH1	NA
	374E@OE2-106R@NE-NE	NA
	374E@OE1-106R@NE-NE	NA
	441N@OD1-130R@HH22-NH2	90.70
	441N@OD1-130R@HH21-NH2	90.70
	441N@OD1-130R@HH12-NH2	88.40
	441N@OD1-130R@HH11-NH1	88.40
	441N@ND2-130R@HH22-NH2	38.20
	441N@ND2-130R@HH21-NH2	38.20

Table S4. Salt bridge percentage in CYP3A5-MDZ complex in two parallel simulations.

Complex CYP3A5-MDZ	The recipient and the donor	ratio
System A	76E@OE1-106R@HH12-NH1	NA
	76E@OE1-106R@HH11-NH1	NA
	76E@OE1-106R@HH22-NH2	14.10
	76E@OE1-106R@HH21-NH2	14.10
	76E@OE2-106R@HH12-NH1	NA
	76E@OE2-106R@HH11-NH1	NA
	76E@OE2-106R@HH22-NH2	10.00
	76E@OE2-106R@HH21-NH2	10.00
	374E@OE2-106R@HH22-NH1	98.90
	374E@OE2-106R@HH21-NH2	98.90
	374E@OE1-106R@HH22-NH2	97.70
	374E@OE1-106R@HH21-NH2	97.70
System B	76E@OE1-106R@HH12-NH1	NA
	76E@OE1-106R@HH11-NH1	NA
	76E@OE1-106R@HH22-NH2	NA
	76E@OE1-106R@HH21-NH2	NA
	76E@OE2-106R@HH12-NH1	NA
	76E@OE2-106R@HH11-NH1	NA
	76E@OE2-106R@HH22-NH2	12.40
	76E@OE2-106R@HH21-NH2	12.40
	374E@OE2-106R@HH12-NH1	27.50
	374E@OE2-106R@HH11-NH1	27.50
	374E@OE1-106R@HH12-NH1	29.50
	374E@OE1-106R@HH11-NH1	29.50

Table S5. The contact number between MDZ and the active residues of the isoforms (5 Å near the active site) in two parallel simulations.

CYP3A4-MDZ	Residues	1st parallel MD	2nd parallel MD
	Phe108	77.64	78.96
	Ala117	86.32	85.56

	Ile120	9.795	5.58
	Phe215	23.56	22.53
	Leu216	25.97	27.22
	Pro218	69.485	68.89
	Phe304	89.35	91.485
	Ala305	84.455	88.38
	Ile369	70.75	68.52
	Ala370	76.95	76.62
	Leu482	21.56	21.03
CYP3A5-MDZ	Leu120	7.705	9.14
	Leu211	6.58	5.36
	Ala297	81.59	79.65
	Val369	80.23	78.65
	Ala370	85.34	82.64
